# Societal burden of work on injury deaths in New Zealand, 2005–14: An observational study

**DOI:** 10.1016/j.ssmph.2023.101353

**Published:** 2023-02-03

**Authors:** Rebbecca Lilley, Gabrielle Davie, Simon Horsburgh, Bronwen McNoe, Tim Driscoll

**Affiliations:** a Preventive and Social Medicine, Dunedin School of Medicine, University of Otago, New Zealand; b Sydney School of Public Health, University of Sydney, NSW, Australia

**Keywords:** Fatal injury, Work, Workers, Commuters, Bystanders, Societal burden, New Zealand

## Abstract

**Background:**

Work poses increased risk of injury not only for workers but also for the public, yet the broader impact of work-related injury is not quantified. This study, utilising population data from New Zealand, estimates the societal burden of work-related fatal injury (WRFI) by including bystanders and commuters.

**Methods:**

This observational study selected deaths due to unintentional injury, in persons aged 0–84 years using International Classification of Disease external cause codes, matched to coronial records, and reviewed for work-relatedness. Work-relatedness was determined by the decedent's circumstances at the time of the incident: working for pay, profit, in kind, or an unpaid capacity (worker); commuting to or from work (commuter); or a bystander to another's work activity (bystander). To estimate the burden of WRFI, frequencies, percentages, rates, and years-of-life lost (YLL) were estimated.

**Results:**

In total 7,707 coronial records were reviewed of which 1,884 were identified as work-related, contributing to 24% of the deaths and 23% of the YLL due to injury. Of these deaths close to half (49%) occurred amongst non-working bystanders and commuters. The overall burden of WRFI was widespread across age, sex, ethnic and deprivation sub-groups. Injury deaths due to machinery (97%) and due to being struck by another object (69%) were predominantly work-related.

**Interpretation:**

When utilising a more inclusive definition of work-relatedness the contribution of work to the societal burden of fatal injuries is substantial, conservatively estimated at one quarter of all injury deaths in New Zealand. Other estimates of WRFI likely exclude a similar number of fatalities occurring among commuters and bystanders. The findings, also relevant to other OECD nations, can guide where public health efforts can be used, alongside organisational actions, to reduce WRFI for all those impacted.

## Highlights

• Close to half of work-related fatalities occurred in groups not typically counted.
• The societal contribution was conservatively estimated at 24% of all fatal injury.
• The burden reaches across society, beyond groups of traditional work concern.
• Work is thus an important, under-recognised source of injury risk across society.
• The widespread burden emphasises the need for multisectoral work and public action.

## 1 Introduction

Work and economic activities have a substantial impact on a range of disease and injury circumstances across society. There are many epidemiological approaches to estimating the burden of work-related harm, with most based on narrow conceptualisations and definitions of work-relatedness, commonly limited to disease and injury in active labour market participants (Driscoll et al., 2005, Driscoll et al., 2005, Leigh et al., 1999; Nelson et al., 2005; Schulte, 2005; Schulte et al., 2017). Few national estimates provide any insight into the broader societal burden of work-related harms, with bystander (exposure of a non-working person to a work risk leading to harm) and commuter cases primarily excluded due to a lack of appropriate data (Driscoll et al., 2005, Driscoll et al., 2005).

This underestimation of the magnitude, dimensionality and societal impact of the burden of work-related disease and injury results in the under-appreciation of the broader societal burden of work-related harm in public health (Driscoll et al., 2005, Driscoll et al., 2005; Schulte et al., 2017). Universal under-recognition of the societal burden of work-related harm means countries miss important opportunities to inform policy and practice from a societal perspective, such as through synergistic or complementary work organisational and public health prevention efforts. Underestimation of the burden of work-related harm directly influences societal investments in worker protection. Broadly inclusive societal data are therefore important to ensure governments, legislators, regulators, insurers, employers, unions, workers, and the public understand the true scale of work-related injury and have the information needed to inform effective control of work-related risks in society (Spieler & Wagner, 2014). Estimates of work-related fatal injury are generally considered to be more comprehensive than those obtained for disease, with clearer connections between work exposures and injury outcomes allowing for comparatively higher proportion of eligible cases to be identified (Driscoll et al., 2005, Driscoll et al., 2005; Schulte et al., 2017).

This study utilised a retrospective national case review of coronial records for injury fatalities to identify cases of WRFI in New Zealand (Lilley et al., 2021; Lilley et al., 2019). Findings from the data, which are broadly applicable to other advanced Organisation for Economic Cooperation and Development (OECD) economies (Driscoll, Marsh, et al., 2005; Feyer et al., 2001), provide the opportunity to obtain a robust population-based estimate of the impact of work on the societal burden of fatal injury, moving beyond including workers to capture other important work-related sub-groups (Lilley et al., 2019). The aim of this study was to identify injury deaths that occurred due to a work-related exposure and to estimate the contribution of work-related exposures to the broader societal burden of fatal injury with the addition of bystanders and commuters.

## 2 Methodology

### 2.1 Study design

A dataset was established of those fatally injured from unintentional incidents in New Zealand from 2005 to 2014. Retrospective case review was undertaken to identify WRFI incidents within this dataset. The study design and methods are described more fully elsewhere and are outlined here (Lilley et al., 2019).

### 2.2 Dataset

Injury deaths due to specified unintentional external causes were obtained from the national Mortality Collection using International Classification of Diseases 10th revision – Australian Modification (ICD-10-AM) underlying cause of death external cause codes (E-codes) within the range V01-X59, and X85-Y34. This range excludes intentional injury due to assault, self-harm, legal intervention, medical misadventure/complications, or war. Injury deaths within the period 2005 to 2014 of those aged 0–84 years were included.

Corresponding coronial records for cases were obtained from the National Coronial Information System (NCIS) (National Coronial Information System, 2016) for the period July 2007 to December 2014 and Archives New Zealand for the period January 2005 to June 2007. For cases obtained from Archives New Zealand, due to the mass storage of coronial records in boxes containing multiple records of interest, all coronial records were physically retrieved regardless of cause of death allowing for the identification of a small number of misclassified cases. It is a legal requirement in New Zealand for all sudden and unexpected deaths to be referred to Coroners for determination of the cause and circumstances of death (New Zealand Government, 2006).

### 2.3 Data sources and measures

The work-relatedness of an injury incident was determined during review of coronial records and was based on whether, at the time of the incident, the decedent was: working for pay, profit or in kind (worker); undertaking work in an unpaid capacity, such as a family farm (worker); commuting directly to or from work (commuter); or a bystander to another person's work activity (bystander) (Lilley et al., 2019). The worker category included a small number of deaths where the decedent was off-duty at the time of the fatal injury incident but there was clear evidence of a cumulative work-related exposure, such as work-related fatigue directly contributing to a traffic crash while not at work. Apprentice and vocational trainees (ie. persons engaged in training with a clear vocational link at the time of injury and thus were analogous to apprentice trainees), were also classified as workers. Bystanders were included regardless of fault as New Zealand has a universal, no-fault injury compensation system that includes both work and non-work injury.

Age, sex, ethnicity, deprivation and external cause of injury were obtained from the Ministry of Health's Mortality Collection. Ethnicity was prioritised into a single ethnic group according to national health data standards in the following order: Māori (indigenous population), Pacific, Asian, and European or Other ethnicities (Ministry of Health, 2017). NZDep is a measure of socio-economic deprivation and was assigned using small areas containing 100–150 people based on usual residential address (Atkinson et al., 2014). NZDep was categorised into quintiles from NZDep 1–2 (least deprived) to NZDep 9–10 (most deprived).

### 2.4 Statistical analysis

**Fig. 1:**
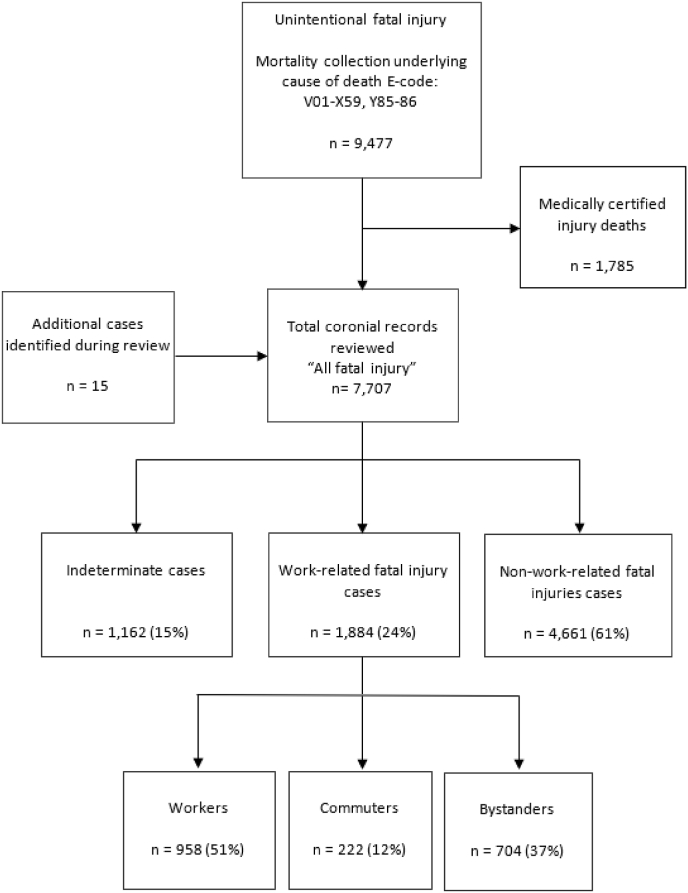
Flow diagram of case selection, deaths due to unintentional injury, age 0–84 years, New Zealand, 2005–2014.

To describe the contribution of WRFI to the societal burden of fatal injuries the number, percentage, and rate per 100,000 population with 95% confidence intervals (CI) were calculated and compared to all injury deaths with a corresponding coronial record (referred to as “all fatal injury” in Fig. 1). Population-based person-years, used as denominators, were calculated overall and by age, sex, ethnicity, and deprivation using estimated annual usually resident counts obtained from Statistics New Zealand.^14^ The proportion of the total burden of all injury occurring at work was calculated as a percentage of all fatal injury cases. To estimate the years of life lost due to work-related death, the years of life lost (YLL) attributable to WRFI exposures and risks were calculated with the remaining life expectancy at the age of death for each case estimated using life tables available from Statistics New Zealand (Statistics New Zealand, 2019). The total YLL was calculated by summing the remaining life expectancy across all cases.

### 2.5 Role of the funding source

The funders of the study had no role in the study design, data collection, analysis, interpretation, or writing of the paper. Access to data was limited to those with NCIS approval (RL, GD, BMcN). All authors had final responsibility for the decision to submit for publication.

## 3 Results

### 3.1 Injury estimates and rates

A total of 9,477 people aged 0–84 years were identified as dying during 2005–2014 from unintentional injury, with an additional 15 initially misclassified cases identified during the manual retrieval and identification of coronial records for review (Fig. 1). A coronial record was available for 7,707 (81%), with this comprising our “all fatal injury” grouping. The remaining medically certified injury deaths (n = 1,785) were considered outside the scope of this study, being predominantly due to the natural progression of disease following fall injuries in the elderly.

### 3.2 Overview of WRFI

The comprehensive review of coronial records identified that at least 1,884 persons had been fatally injured due to a work-related exposure. Bystanders and commuters constituted a substantial proportion of the deaths attributed to work exposures, accounting for at least 49% (95% CI 47,51) of the burden of WRFI: bystanders, 37% and commuters, 12% (Fig. 1).

The societal rate of WRFI was 4·4 deaths per 100,000 person-years (Table 1). The rate of WRFI increased with increasing age to peak at 6·3 deaths per 100,000 person-years for those aged 55–64 years. Males had a rate of WRFI 3·5 times higher than that for females. Variation in rates of fatal injury by ethnicity are apparent for both all-cause and WRFI. Rates of WRFI by deprivation were relatively similar (varied by 30% or less) compared to the strong linear gradient apparent in all fatal injury (rate for NZDep 9-10) was double that of NZDep 1–2.

**Table 1:** Number, Percentage and Rate of All and Work-Related Fatal Injury Among the Population aged 0–84 years, by socio-demographic and injury cause, New Zealand, 2005–2014 (per 100,000 population).

	All injury^1^				Work-related injury				Proportion occurring at work^3^ (%)
	Number (n)	Percentage (%)	Rate^2^	95% CI	Number (n)	Percentage (%)	Rate^2^	95% CI	
Age (years)									
0-14	680	9	7.5	6.9, 8.1	101	5	1.1	0.9, 1.3	15
15-24	1484	19	24.1	22.9, 25.4	316	17	5.1	4.5, 5.7	21
25-34	971	13	17.8	16.6, 18.9	267	14	4.9	4.3, 5.5	27
35-44	1185	15	19.2	18.1, 20.3	321	17	5.2	4.6, 5.5	27
45-54	1195	16	19.8	18.7, 21.0	338	18	5.6	5.0, 6.2	28
55-64	933	12	19.7	18.4, 21.0	300	16	6.3	5.6, 7.1	32
65-84	1259	16	25.3	23.9, 26.7	241	13	4.8	4.2, 5.5	19

Sex									
Female	2203	29	10.2	9.7, 10.6	428	23	2.0	1.8, 2.2	19
Male	5504	71	26.3	25.6, 27.0	1456	77	7.0	6.6, 7.3	26

Ethnicity									
Māori	1829	24	27.8	26.5, 29.1	374	20	5.7	5.1, 6.3	20
Pacific peoples	368	5	13.6	12.2, 15.1	65	3	2.4	1.8, 3.1	18
Asian	448	6	9.9	9.0, 10.8	111	6	2.5	2.0, 2.9	25
European & Other	5050	66	17.6	17.1, 18.1	1331	71	4.6	4.4, 4.9	26
Missing	12	0			3	0			25

Deprivation (NZ Dep)									
1 to 2 (least)	1011	13	12.0	11.3, 12.8	310	16	3.7	3.3, 4.1	31
3 to 4	1151	15	14.0	13.2, 14.8	290	15	3.5	3.1, 3.9	25
5 to 6	1273	17	15.8	14.9, 16.7	373	20	4.6	4.1, 5.1	29
7 to 8	1578	20	19.8	18.8, 20.8	346	18	4.4	3.9, 4.8	22
9 to 10 (most)	1930	25	24.0	22.9, 25.1	349	19	4.3	3.9, 4.8	18
Missing	764				216				

Total	7707		18.1	17.7, 18.5	1884		4.4	4.2, 4.6	24

Key: 1 - Corresponds to Total Coronial records reviewed as indicated in Figs. 1, 2 – Rate per 100,000 person/worker years, 3 - Minimum proportion of all injury burden occurring at work.

### 3.3 Contribution of non-workers to burden

Appendix 1 presents the characteristics of WRFI by work circumstance. Non-working bystanders were mainly killed in work-related transport events; the 614 such deaths represent 87% of the bystanders identified for the period. The most common transport scenario was head-on traffic collisions involving a commercial working vehicle, such as a truck, and a driving member of public in a light vehicle on unseparated dual carriage State Highway. Combining bystander and commuter deaths demonstrates the substantial burden that these deaths add to the total burden of WRFI. In total they comprise 21% (833/4,059) of all fatal unintentional injury and 65% (833/1,280) of all WRFI due to transport mechanisms alone. Notably high proportions of bystander deaths include those aged 0–24 years (0–14 years, 93% and 15–24 years, 52%), females (59%), those of Asian ethnicity (51%) and those fatally injured due to drowning (50%).

### 3.4 Contribution of work to unintentional fatal injury

Of the 7,707 fatalities reviewed, 1,884 (24%) sustained fatal unintentional injuries as the result of a work-related exposure (Fig. 1). The estimated 24% is a minimum bound as it was not possible to determine whether the incident was a WRFI case, or not, for 1,162 (15%) cases (Fig. 1). Indeterminant cases were dominated by road traffic crashes: 91% involved traffic crashes on public roads, where the purpose of a trip was unable to be determined from the coronial record.

The contribution of work to the burden of all fatal injury was at least 15% in all age groups, peaking at 32% of all fatal injury in those aged 55–64 years (Table 1). Of those for which work-relatedness could be determined, one in four injury deaths in males was attributed to work exposures, compared to one in five deaths for females. One quarter of injury deaths in those of either European or Asian ethnicity were also attributed to work causes, while work contributed to at least 20% of unintentional injury deaths for indigenous Māori.

Generally, the contribution of work was lowest in those living in areas with the highest levels of deprivation (NZ Dep 7–8, 22% and NZ Dep 9–10, 19%). The highest contribution of work to fatal injury was observed in those in the lowest deprivation quintile (NZ Dep 1–2, 31%) and the medium quintile (NZ Dep 5–6, 29%) (Table 1).

The contribution of work varied by mechanism of fatal injury (Table 2). Virtually all deaths caused by machinery were attributed to work exposures, with at least 97% of all such fatalities considered to be a WRFI. Other external causes of injury deaths with notable levels of work involvement include those caused by being struck by, or against, another object (69% work-related), and those due to natural/environmental causes (41% work-related). As they are for all fatal injuries, transport mechanisms are an important cause of WRFI, accounting for 68% (n = 1280) of all WRFI. Close to one in three transport deaths were attributed to work. The smallest contribution of work occurred for fatal injuries caused by acute poisonings, where only 1% of fatalities were due to a work-related exposure.

**Table 2:** Number and Percentage All and Work-Related Fatal Injury Among the Population aged 0–84 years, by injury cause (ICD-10 categories), New Zealand, 2005–2014

Mechanism of injury	All injury		Work-related injury		Proportion of all injury burden occurring at work
	Number (n)	Percentage (%)	Number (n)	Percentage (%)	
Cut/pierce	27	0.3	8	0.4	30
Drowning	546	6.4	37	1.9	7
Fall	790	9.3	94	4.9	12
Fire	172	2.0	8	0.4	5
Firearms	24	0.3	7	0.4	29
Machinery	113	1.3	110	5.7	97
Natural/Environ	293	3.5	121	6.3	41
Other	249	2.9	98	5.1	39
Poison	882	10.4	12	0.6	1
Struck by/against	116	1.4	80	4.1	69
Suffocation	421	5.0	14	0.7	3
Transport	4,082	48.1	1280	66.3	31
Missing	15	0.2	15	0.8	100

### 3.5 Years of life lost (YLL) due to work

Of all fatal injury in New Zealand for the decade, an estimated 86,682 years of life, or 23% of the total burden of YLL, was attributable to WRFI including bystanders and commuters (Table 3). Of the YLL lost due to WRFI, 54% occurred in non-workers (total 47,051, mean 51.6) and 46% (total 39,631, mean 41.4) occurred in workers. By extension, WRFI in non-workers contribute to 13% of the YLL and workers contribute to 11% of the YLL lost to all fatal injury.

**Table 3:** Years of life lost for All and Work-related injury among the New Zealand population aged 0–84 years, 2005–2014.

	All injury		Work-related injury		Proportion of all injury YLL occurring due to work (%)
	Total	Mean	Total	Mean	
Sex					
Male	264661	48.08	65846	45.22	25
Female	104416	47.39	20836	48.68	20

Ethnicity					
Maori	106077	57.99	19203	51.34	18
Pacific peoples	21087	57.30	3235	46.76	24
Asian	23668	52.83	5661	51.00	15
European & other	217582	43.08	58450	43.91	27
Unspecified	633		133		

Deprivation (NZDep)					
1 to 2 (least)	44512	44.02	13624	43.94	31
3 to 4	53875	46.81	13166	45.40	24
5 to 6	59305	46.58	16781	44.99	28
7 to 8	75190	47.64	16142	46.65	21
9 to 10 (most)	100245	51.94	17114	49.03	17
Unspecified	35950		9855		

Underlying cause (ICD-10)					
Cut/pierce	1203	44.55	276	34.55	23
Drowning	28445	52.09	2274	61.47	8
Fall	24687	31.25	3208	31.13	13
Fire	7272	42.28	224	28.07	3
Firearms	1172	48.84	274	39.15	23
Machinery	4770	42.21	4689	42.63	98
Natural/Environ	14327	48.89	5675	46.90	40
Other	11006	41.94	4709	42.30	40
Poison	40337	45.73	494	41.24	1
Struck by/against	5393	46.49	3373	42.17	63
Suffocation	27419	65.12	693	49.56	3
Transport	203046	50.02	60793	47.49	30

Total	369077	47.89	86682	46.00	23

Notably high levels of YLL lost due to WRFI included those fatally injured by machinery (98% of YLL lost due to machinery mechanisms), being struck by/against an object (63%), and natural/environmental (40%) hazards (Table 3). Work contributed to 31% of YLL lost amongst those living in the least deprived areas (NZ Dep 1–2).

## 4 Discussion

The contribution of work to the broader societal burden of fatal injury in New Zealand is substantial. With the inclusion of bystanders and commuters this study conservatively estimated 24% of all fatal injury investigated by Coroners and 23% of the aggregated loss of life due to all fatal injury was work-related, identifying for all stakeholders the burden of WRFI that must be managed. The total burden of WRFI was found to be widespread across the population, commonly occurring across all age, sex, and ethnic sub-groups, as well as all levels of deprivation and injury mechanisms. This indicates that work-related exposures are an important and under-recognised source of injury risk across society.

To the best of our knowledge this is the first study to estimate the contribution of WRFI to the societal burden of fatal injuries on a national basis. A pilot study from one state in Australia similarly estimated 21% of unintentional fatal injury was work-related (Bugeja et al., 2009). Estimates of non-fatal injury based on population-based survey data in the United Kingdom and the United States suggest around 29% of the total injury burden was work-related (Smith et al., 2005; Wilkins & Mackenzie, 2007) and report similarities in etiological characteristics between work and non-work injury morbidity (Smith et al., 2006). Global Burden of Disease (GBD) figures based on ICD classifications of external causes of injury estimate unintentional occupational injury in workers alone contributed to a minimum of 8·8% of the global burden of mortality due to unintentional injury in 2000 (Concha-Barrientos et al., 2005). More recent estimates of GBD for 2016 estimate occupational risk factors, account for 2·8% of deaths and 3·2% of DALYs from all causes. (GBD 2016 Occupational Risk Factors Collaborators, 2020) It is well recognised that most national and global measures of occupational injury and disease are underestimates due to inadequate data, and definitional and measurement issues (GBD 2016 Occupational Risk Factors Collaborators, 2020; Concha-Barrientos et al., 2005; Spieler & Wagner, 2014). The need for additional estimates to provide a broader societal measure of the burden of WRFI is clear as current measures limit the scope of safety policy and actions, constraining where resources are placed and the attention given to this issue.

Our study found that bystanders make a substantial contribution to the burden of WRFI in society and impact widely across sociodemographic and injury risks present in the community. Despite observed variations in the relative contribution of WRFI by age, work exposures importantly influence the burden of fatal injury across the life course, supporting an expanded WRFI prevention focus to include children, youth, and older persons. Also, while WRFI was found to be more prevalent overall in males, females represent a greater proportion of bystander deaths, making them an equally important hidden group to target for preventive action from an equity perspective. Understanding differences in ethnic, indigenous and deprivation groups in WRFI is of political and societal importance. This study found WRFI was experienced across all ethnic groups and all levels of deprivation pointing to the need to prioritise the prevention of WRFI to contribute to reductions in the overall burden of injury, particularly for sub-groups with much higher overall rates of fatal injury.

Our study found bystander and commuter fatalities were predominantly due to transport-related causes, occurring on public roads, demonstrating the high risk of WRFI due to public exposure to working vehicles, and the hazards they pose, on public roads. Within national data collections much of the burden of WRFI on public roads is currently, at best, under-recognised for workers and commuters, and commonly excludes groups like bystanders who are indirectly exposed to working vehicles (Lilley et al., 2013; Murray et al., 2008). The limited research enumerating the contribution of non-working circumstances to work-related injury also points to work-traffic fatalities involving a large number of bystanders: 49% in Australia and 85% in Ireland (Drummond et al., 2016; Mitchell et al., 2004). Our current estimate of bystander involvement in at least 37% of the WRFI burden represents a reduction from previous New Zealand estimates of 52% of the WRFI burden from 1985 to 1998 (Langley et al., 2006). Despite the reduction in the relative contribution of bystanders to the overall burden of WRFI, the proportion of bystander deaths due to work-related traffic crashes has remained consistent with around three quarters of bystander deaths due to work-traffic events for both periods (Langley et al., 2006).

In New Zealand, organisations are mandated to manage and control the risks associated with work journeys on public roads under their organisational influence. Increased understanding and recognition of the spill-over of work-related risks onto public roads will support the identification of those risks to be addressed in population-focused strategies with synergistic potential to jointly address the burden of transport-related fatalities more broadly. Such effective population-based road safety strategies, controls and improvements could include the adoption of stricter vehicle safety standards, public enforcement of occupant restraint use, and improved integration of key safety road design features, such as median division or wide roadside shoulders, in upgrades of national roading infrastructure (Peden et al., 2004).

The potential benefits of a public health approach are largely overlooked in occupational health and safety (Ahonen et al., 2018; Breslin & Shannon, 2005; Smith, 2001). While concepts of work-related harm evolve, definitions often focus on operational aspects of occupational health and safety legislation, and are differentiated from other systems of injury control (Allen & Clarke, 2009). As a result the interface between public health and occupational health and safety is not strong, despite the costs of this harm largely being borne by workers, their families, and public health and welfare systems. (Access Economics, 2006) Our findings indicate the adoption of a public health approach, in addition to organisational controls, is warranted in areas such as public roads, where there are shared risks and benefits of a population-based strategy. Work-related injury risks that predominantly occur under the responsibility and control of organisations, including risks posed by machinery, moving objects and the natural environment, should remain the primary responsibility of organisations.

This study has several important strengths. The comprehensive dataset established through the systematic review of population based coronial records, originally identified from national mortality records, providing a measure of absolute risk is a clear strength of this study. The robust methodology employed on a single source of population data allows for estimation of the societal burden through the application of broader conceptualisations of work, thus overcoming the traditional political and legislatively constrained definitions of work-relatedness applied to official data collections. Additionally, these data capture WRFI across the life course up to 85 years of age, meaning the estimates obtained in this analysis are based on a more comprehensive data source than available elsewhere. These data are broadly generalisable to other advanced economy OECD countries. Assuming similar contributions in other nations, a greater focus on preventing WRFI could save millions of lives and contribute to substantive reductions in the burden of injury across the world.

There are some limitations to the estimates presented here. Estimates for deaths in the work traffic setting are likely to be conservative, due to difficulties in identifying cases in circumstances involving light vehicles, such as cars, where the purpose of journey is often not described in coronial records and is therefore unable to be determined (e.g. 15% of cases had insufficient information to determine work-relatedness, of which 91% involved traffic crashes on public roads). Some misclassification was identified, although it was identified as a very small number of 15 cases being classified in the application of the ICD classification system to a natural cause, such as septicaemia following a penetrating injury, rather than an external cause during case review. The WRFIS estimates in this study are limited to fatal injuries with unintentional intent, so is missing deaths due to intentional assaults or self-harm, which constitute one third of deaths due to external causes in New Zealand (Injury Prevention Research Unit, 2021). Review was limited to deaths reported to and investigated by Coroners. Despite the compulsory notification of all deaths “without known cause, or self-inflicted, unnatural, or violent” to Coroners in New Zealand (New Zealand Government, 2006), 18% of injury deaths in the national Mortality Collection were not the subject of a coronial investigation due to being medically certified deaths. Most medically certified cases are unlikely to be work-related, with 70% of these cases aged over 70 years and dying due to a fall; therefore, the impact of any selection biases on estimates of WRFI are likely to be small. The prolonged length of time to close coronial investigations limits the currency of the data. Coronial records, however, provide the best data available to independently ascertain the likely involvement of work across a range of injury causes and circumstances.

It is hoped that this paper will stimulate debate on the pressing need for improvements in the work-related representation of WRFI data to increase focus on the control and management of work-related injury risks. Socially inclusive estimates of the population at risk of harm from work-related hazards that also include commuters and bystanders would stimulate responsive preventive action better able to manage societal injury risks posed by work and economic activity. Cohesive national and international WRFI surveillance efforts are therefore needed to establish more accurate and inclusive societal estimates of burden. These estimates of burden, combined with analyses (e.g. age-sex standardised rates) that focus on identifying subgroups at higher risk of WRFI should be used to inform evidence-based policies and actions, and to strengthen understanding of the contribution of work to the broader societal burden of injury. The current lack of detailed empirical data provides little guidance as to where complementary or synergistic organisational and population-based safety efforts might be best planned and implemented. Future data system improvements to broaden the conceptualisation of work-relatedness could focus on utilising free text analysis, increasing the range of population-based health and administrative datasets capturing work-relatedness, and adopting new means of investigating and capturing work involvement at the population level (Ahonen et al., 2018; Smith, 2001).

In conclusion, in utilising a more socially inclusive definition of work-relatedness, this study conservatively estimated at least a quarter of injury deaths in New Zealand were work-related, with close to half these deaths occurring in groups not typically counted in estimates of WRFI. This study shows that work-related risks are an important yet under-recognised determinant of injury and emphasises the overlap between occupational and public health risks in terms of fatal injury. The findings, also relevant to other OECD nations, serve to highlight the importance of capturing more socially inclusive estimates of the burden of WRFI to guide where public health efforts can be used, alongside organisational actions, to reduce WRFI for all those impacted: workers, commuters, and bystanders.

## Funding

This work was supported by the 10.13039/501100001505Health Research Council of New Zealand (ref HRC 16/173).

## Data sharing

Data may be obtained from a third party. The primary data used for this study were obtained from Archives New Zealand, Ministry of Justice and the National Coronial Information System administered by a third parties. These data are not publicly available.

## Author statement

RL led all aspects of the study, including conceptualisation, funding acquisition, project administration, investigation, methodology, formal analysis, and wrote the original draft of the article. All authors contributed to the conceptualisation, funding acquisition, methodology discussion and interpretation of the results, reviewing and editing subsequent drafts of the article. GD additionally contributed to data curation, project administration and formal analysis, while BMcN contributed to project administration and investigation. All authors have read and approved the final manuscript.

## Patient and public involvement

This study involves deceased persons therefore patients and public were not involved in the design, conduct, reporting or dissemination plans of this study.

## Ethical approvals

Ethical approval for this study was granted by the University of Otago Human Ethics Committee (Ref 15/065) and for the use of the relevant data by the National Coronial Information System (Ref NZ007), and Health and Disability Ethics Committee (Ref OTA/99/02/008/AM05).

## Declaration of competing interest

None.

## Data Availability

The authors do not have permission to share data.
