# Supplementary material for: Societal burden of work on injury deaths in New Zealand, 2005–14: An observational study
Source: SSM Popul Health. 2023 Feb 3;21:101353. doi: 10.1016/j.ssmph.2023.101353 (PMC9945765; doi:10.1016/j.ssmph.2023.101353)
Supplement: Multimedia component 1 [file mmc1.docx]

Appendix 1. Number and Percentage Work-Related Fatal Injury by Work Circumstance, New Zealand, 2005-2014

|  | | **Work Circumstance** | | | | | | | |
| --- | --- | --- | --- | --- | --- | --- | --- | --- | --- |
|  |  | **Workers** | |  | **Bystander** | |  | **Commuters** | |
|  |  | **Number (n)** | **Percentage (%)** |  | **Number (n)** | **Percentage (%)** |  | **Number (n)** | **Percentage (%)** |
| Age (years) | |  |  |  |  |  |  |  |  |
|  | 0-14 | 7 | 1 |  | 94 | 14 |  | 0 | 0 |
|  | 15-24 | 101 | 11 |  | 164 | 23 |  | 51 | 23 |
|  | 25-34 | 139 | 15 |  | 91 | 13 |  | 37 | 17 |
|  | 35-44 | 190 | 20 |  | 92 | 13 |  | 39 | 17 |
|  | 45-54 | 208 | 21 |  | 79 | 11 |  | 51 | 23 |
|  | 55-64 | 196 | 20 |  | 73 | 10 |  | 31 | 14 |
|  | 65-84 | 117 | 12 |  | 111 | 16 |  | 13 | 6 |
|  |  |  |  |  |  |  |  |  |  |
| Sex | |  |  |  |  |  |  |  |  |
|  | Female | 99 | 10 |  | 258 | 37 |  | 71 | 32 |
|  | Male | 859 | 90 |  | 446 | 63 |  | 151 | 68 |
|  |  |  |  |  |  |  |  |  |  |
| Ethnicity | |  |  |  |  |  |  |  |  |
|  | Māori | 173 | 18 |  | 159 | 23 |  | 42 | 19 |
|  | Pacific peoples | 25 | 3 |  | 23 | 3 |  | 17 | 8 |
|  | Asian | 38 | 4 |  | 60 | 9 |  | 13 | 7 |
|  | European & Other | 720 | 75 |  | 462 | 65 |  | 149 | 66 |
|  | Missing | 2 | 0 |  | 0 | 0 |  | 1 | 0 |
|  |  |  |  |  |  |  |  |  |  |
| Deprivation (NZ Dep) | | |  |  |  |  |  |  |  |
|  | 1 to 2 (least) | 187 | 20 |  | 100 | 14 |  | 23 | 11 |
|  | 3 to 4 | 163 | 17 |  | 89 | 12 |  | 38 | 17 |
|  | 5 to 6 | 186 | 19 |  | 126 | 18 |  | 61 | 27 |
|  | 7 to 8 | 179 | 19 |  | 131 | 19 |  | 36 | 16 |
|  | 9 to 10 (most) | 155 | 16 |  | 148 | 21 |  | 46 | 21 |
|  | Missing | 88 | 9 |  | 110 | 16 |  | 18 | 8 |
|  |  |  |  |  |  |  |  |  |  |
| Mechanism of injury | | |  |  |  |  |  |  |  |
|  | Cut/pierce | 8 | 1 |  | 0 | 0 |  | 0 | 0 |
|  | Drowning | 16 | 2 |  | 20 | 3 |  | 0 | 0 |
|  | Fall | 74 | 8 |  | 20 | 3 |  | 0 | 0 |
|  | Fire | 7 | 1 |  | 1 | 0 |  | 0 | 0 |
|  | Firearms | 5 | 1 |  | 2 | 0 |  | 0 | 0 |
|  | Machinery | 103 | 10 |  | 7 | 1 |  | 0 | 0 |
|  | Natural/Environ | 96 | 10 |  | 24 | 3 |  | 0 | 0 |
|  | Other | 95 | 10 |  | 9 | 1 |  | 0 | 0 |
|  | Poison | 11 | 1 |  | 1 | 0 |  | 0 | 0 |
|  | Struck by/against | 74 | 8 |  | 6 | 1 |  | 0 | 0 |
|  | Suffocation | 14 | 1 |  | 0 | 0 |  | 0 | 0 |
|  | Transport | 447 | 45 |  | 618 | 86 |  | 219 | 97 |
|  | Missing | 9 | 1 |  | 5 | 1 |  | 1 | 0 |
|  |  |  |  |  |  |  |  |  |  |
| Total | | 958 |  |  | 704 |  |  | 222 |  |
